# Right Lower Extremity Phlegmasia Cerulea Dolens Due to Iliotibial Vein Thrombosis With Compartment Syndrome and Circulatory Shock: Case Report and Review

**DOI:** 10.7759/cureus.32364

**Published:** 2022-12-09

**Authors:** Kingsley Dah, Ikechukwu R Ogbu, Chinedu Ngwudike, Maanas Tripathi

**Affiliations:** 1 Internal Medicine, Texas Tech University Health Sciences Center El Paso, Paul L. Foster School of Medicine, El Paso, USA; 2 Cardiology, Sunrise Health Graduate Medical Education Consortium, Las Vegas, USA; 3 Internal Medicine, HCA Las Palmas Del Sol Healthcare, El Paso, USA

**Keywords:** circulatory shock, fasciotomy, multiorgan failure, compartment syndrome, deep vein thrombosis, phlegmasia cerulean dolens

## Abstract

Phlegmasia cerulea dolens (PCD) is a rare and life-threatening complication of extensive deep vein thrombosis (DVT) characterized by severe pain, swelling, and cyanosis of the affected limb. It results from total or near-total occlusion of the deep and superficial veins of a limb, leading to venous congestion and ischemia. It is associated with 40% mortality, more commonly affecting the left lower extremity, with up to 50% of patients requiring limb amputations. PCD complicated by compartment syndrome (CS) with shock and multiorgan failure is very rare. We report the case of a 55-year-old female who presented with sudden onset, severe right lower extremity pain and swelling with associated limb discoloration, paresthesias, and inability to move the toes of her right foot. On examination, there was cyanosis, pulselessness, and tense right leg and thigh compartments. Doppler ultrasonography revealed DVT of the right external iliac extending to the posterior tibial vein. A diagnosis of PCD with CS was made and the patient was immediately started on anticoagulation with unfractionated heparin and emergent decompressive fasciotomies of the right leg and thigh were performed. Following the fasciotomies, she developed circulatory shock and went into cardiac arrest. Despite successful resuscitation, her hemodynamic instability and multiorgan failure precluded further life-saving interventions including thrombolysis or thrombectomy. Limb amputation was declined given her poor prognosis and she passed away shortly thereafter. This case illustrates the rare occurrence of right lower extremity PCD complicated by CS, circulatory shock, and multiorgan failure, which can sometimes occur despite emergency fasciotomy but can be averted with prompt intervention. These complications often preclude immediate thrombolysis and/or thrombectomy. Its recognition, therefore, warrants timely and more aggressive interventions to prevent limb loss or death.

## Introduction

Phlegmasia cerulea dolens (PCD) is a rare and life-threatening complication of acute deep vein thrombosis (DVT) resulting from an extensive total or near-total occlusion of the deep and superficial venous drainage systems of a limb. It is associated with up to 40% mortality while up to 50% of presenting patients may require limb amputation [1]. Malignancy remains the most common risk factor, seen in 20-40% of cases, with slightly more males than females being affected, and the left lower extremity up to four times more likely to be affected than the right [1,2].

PCD is characterized by severe and fulminant pain, swelling, and cyanosis of the affected limb. It represents an advanced stage in the spectrum of acute DVT complications from early phlegmasia alba dolens (painful, inflamed white limb) to venous gangrene. Unlike phlegmasia alba dolens, in which thrombosis only involves deep veins allowing for drainage via the unaffected superficial and collateral veins, PCD involves a complete occlusion of the major and collateral veins, resulting in venous congestion, fluid sequestration, arterial insufficiency, and ischemia [3]. Without prompt intervention, this can progress to venous gangrene and compartment syndrome (CS) with resulting metabolic and hemodynamic deterioration that often leads to loss of limb or death. Although uncommon, PCD with CS has been previously reported [1,4]. However, PCD of the right lower extremity is complicated by CS, and multiorgan failure is rare [5]. We report a case of a right lower extremity PCD complicated by the development of CS and circulatory shock with multiorgan failure.

## Case presentation

A 55-year-old female presented to the emergency department (ED) with sudden onset severe right lower extremity pain with swelling. She had first noticed swelling of her right lower extremity with no associated pain or motor deficits about three to four days prior to presentation. The pain and swelling initially started distally but rapidly progressed more proximally towards her thigh, with associated limb discoloration and paresthesias. Her past medical history included a recently diagnosed stage I right breast cancer for which she was not receiving treatment, hypertension, diabetes mellitus, and uterine fibroids. Her only medications were metformin and oral contraceptives. There was no history of trauma, immobility, recent surgery, long-distance travels, venous thromboembolism (VTE), or personal or family history of bleeding, or clotting disorders.

Upon initial examination, her blood pressure (BP) was 149/71 mmHg, with a heart rate of 101/min, a respiratory rate of 16/min, a temperature of 36.4 ^o^C, and blood oxygen saturation (SpO2) of 94% on room air. The right thigh and leg were edematous with diffuse purple discoloration, tense thigh and leg compartments with no palpable posterior tibial and dorsalis pedis pulses. The patient had loss of sensation below the knee with an inability to move the toes of her right foot or actively perform a straight leg raise. Any attempted passive range of motion was extremely painful and out of proportion to the exam. The left lower extremity, however, was normal with no swelling, tenderness, or motor, or sensory deficits and all compartments were soft with palpable distal pulses.

**Figure 1:**
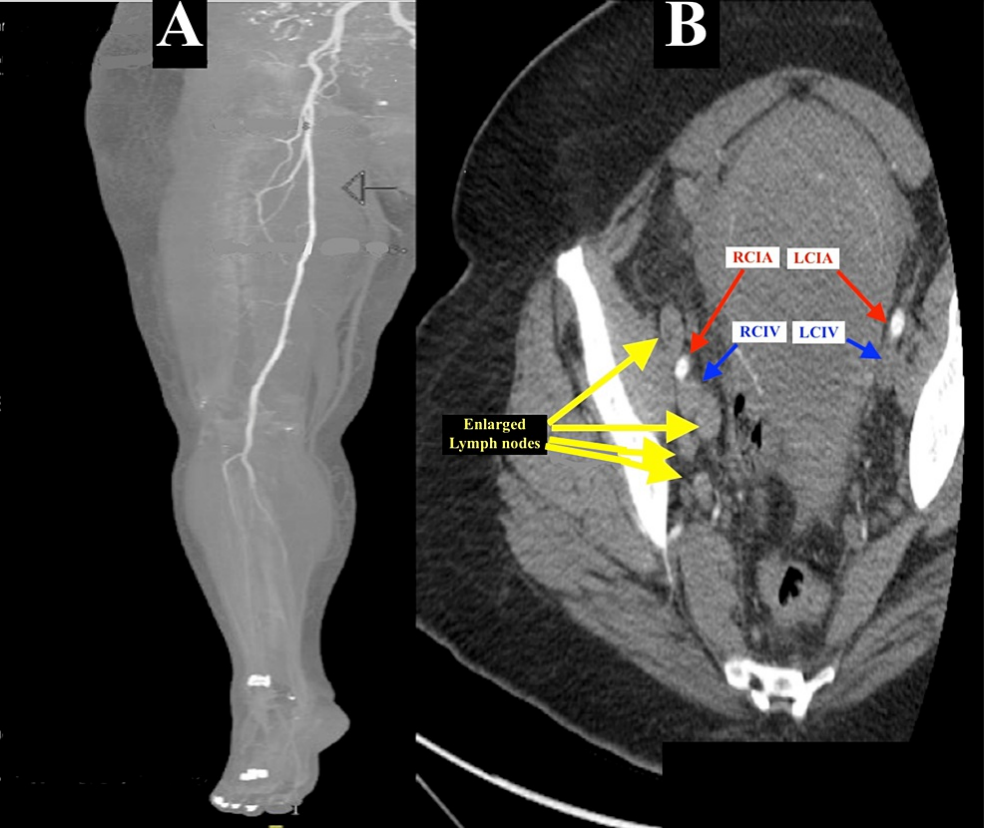
Computed tomography angiography of the right lower extremity and pelvis (A-B): Enlarged iliac chain lymphadenopathy worse on the right than left (yellow arrows), along the right pelvic sidewall abutting the right common iliac artery (red arrow) and compressing an enlarged iliac vein (blue arrow), effacing the right external iliac vein and likely causing the iliotibial DVT distally (not shown). Notice the significant edema and swelling of the right thigh and leg (A) with no arterial occlusive disease (grey arrow). DVT: deep vein thrombosis; RCIA: right common iliac artery; LCIA: left common iliac artery; RCIV: right common iliac vein; LCIV: left common iliac vein.

Initial laboratory findings were significant for leukocytosis with white blood cell count (WBC) of 22.7 (4.00 -10.5 x 10^3^ /mm^3^ ), normocytic anemia with hemoglobin (Hgb) of 9.4 (13.0 - 17.5 g/dL), hypokalemia with a potassium level (K) of 2.8 (3.5 - 5.1 mmol/L), high anion gap metabolic acidosis with a bicarbonate level (HCO_3_) of 19 (21 - 32 mmol/L), anion gap of 17 (normal range < 12), elevated lactate at 2.8 (0.5 - 1.9 mmol/L) which improved to 1.6 mmol/L with intravenous fluid hydration, and a normal renal function with a blood urea nitrogen (BUN) of 15 (7 - 18 mg/dL) and serum creatinine (Cr) of 0.8 (0.6 - 1.3 mg/dL). Venous ultrasonography of the right lower extremity showed a totally occluded and non-compressible right external iliac vein, extending up to the posterior tibial veins. A computed tomography angiography (CTA) of the right lower extremity showed no arterial occlusive disease but there was an enlarged right iliac adenopathy which appeared to be compressing the right external iliac vein (Figure 1).

Based on the patient’s clinical presentation of severe right lower extremity pain, swelling, and cyanosis, coupled with evidence of extensive right lower extremity iliotibial DVT, a diagnosis of PCD was made. She was immediately started on anticoagulation with unfractionated heparin (UFH) infusion, fluid hydration, and pain control. Given the development of tense right thigh and leg compartments with no palpable distal pulses, orthopedic surgery was consulted for concern of a developing CS. Upon evaluation and noting the ensuing CS, the patient was promptly taken to surgery for emergency decompressive fasciotomies to relieve the pressure in the right thigh and leg compartments. Interventional radiology and vascular surgery were also consulted for thrombolysis or thrombectomy for the extensive iliotibial DVT, however, this was deferred in favor of the emergency fasciotomies for the CS. Leg fasciotomies revealed some dusky-appearing muscles in the superficial and deep posterior compartments, but there was no necrosis, and contractility was intact. Thigh fasciotomies demonstrated well-perfused muscle tissue. The estimated blood loss (EBL) during fasciotomies was about 300 ml and the patient received two units of packed red blood cells transfusion during the surgery. At the end of the surgery, bilateral distal pulses were elicited by Doppler ultrasound, with improved warmth to the right foot, and the patient was able to move her toes again. She was then transferred to the intensive care unit for postoperative resuscitation and management with close monitoring of anticoagulation. Catheter-directed thrombolysis/thrombectomy was planned for the following morning after the fasciotomies.

About six hours after surgery the following morning, the patient developed circulatory shock as she became hypotensive (BP of 80/52), tachypneic, and tachycardic with altered mentation, requiring vasopressor support and intubation with mechanical ventilation for airway protection and hypoxemia. Repeat laboratory findings at that time showed worsening leukocytosis (WBC 31), anemia (Hgb 8), metabolic acidosis (HCO_3_ 5.7), hyperkalemia (K 6.3), acute renal failure (BUN 22, Cr 1.7) with oliguria, elevated creatine kinase levels at 1749 (26 - 192 U/L), and shock liver with elevated aspartate transaminase (AST) at 1611 (15 - 37 U/L) and elevated alanine transaminase (ALT) at 1635 (10 - 49 U/L). Arterial blood gas analysis prior to intubation was consistent with hypoxemia and metabolic acidosis: pH 7.06 (7.35 - 7.45), partial pressure of carbon dioxide (pCO2) of 20 (35 - 45 mmHg), and partial pressure of oxygen (pO2) of 52 (80 - 100 mmHg).

Given the worsening metabolic acidosis and hyperkalemia which was refractory to medical therapy, nephrology was consulted, and renal replacement therapy was initiated. The patient, however, was too unstable to complete the initial planned two-hour dialysis session, so it was stopped prematurely. Soon afterward, she had a cardiac arrest due to ventricular fibrillation (rhythm at the time of arrest). Two cycles of cardiopulmonary resuscitation with defibrillation, intravenous bicarbonate, and calcium gluconate treatments were completed with a successful return of spontaneous circulation and sinus rhythm. At this point, given her hemodynamic instability and multiorgan failure, the planned catheter-directed therapies or thrombectomy were deferred. Limb amputation was also explored but declined by the patient's family given her overall grim prognosis. Per the patient’s next of kin’s request, all supportive measures were eventually withdrawn, and the patient was pronounced dead shortly thereafter, within 24 hours of presentation.

## Discussion

Given our patient’s presentation with worsening right lower extremity swelling, cyanosis, and pain, complicated by the development of CS and acute deterioration into shock within 24 hours, differential diagnoses other than PCD include complicated cellulitis, venous gangrene, arterial occlusive disease and purpura fulminans (PF). On initial evaluation, the patient’s limb was not gangrenous, however, it was quickly progressing to venous gangrene as CS set in. She was also afebrile and vitally stable without hemodynamic compromise, only going into shock several hours later, after the fasciotomies. Additionally, a right lower extremity CTA showed no acute occlusive disease. Interestingly, PF, which can have a similarly dramatic and often life-threatening presentation as PCD, is a rare condition characterized by widespread thrombosis of small blood vessels resulting in disseminated intravascular coagulation (DIC) with hemorrhagic skin infarction and necrosis, manifesting as retiform purpura of the affected skin areas, and sometimes complicated by shock, multiorgan failure, and death [6-8].

Although commonly seen in children, PF can occur in adults and is often infectious in origin, commonly due to disseminated encapsulated bacterial infections from *Streptococcus pneumoniae*, *Neisseria meningitidis*, *Haemophilus influenzae*, among other pathogens, which mediate cytokine cascades leading to coagulation dysregulation and endothelial damage often manifesting as DIC, sepsis and/or shock [7, 8]. The features of infectious PF often include the signs and symptoms of the underlying infection, followed by DIC with a widespread purpuric rash which might start as macules and progress into bullae [6, 8]. Our patient did not report any recent illnesses or rashes, was afebrile and hemodynamically stable on presentation, and no purpura or bullae were evident on exam. PF was therefore low on our list of differentials. One could make a case for a possible underlying infection on admission given the leukocytosis, elevated lactate, and tachycardia on presentation, concerning for systemic inflammatory response syndrome or sepsis. These findings, however, can also be seen in a patient with PCD developing CS, with ensuing venous congestion, arterial insufficiency, and ischemia. Additionally, there was no demonstrable abscess or subcutaneous gas on right lower extremity imaging, and blood cultures drawn on admission yielded no growth. Nonetheless, an infectious component could not be excluded later in her hospital course when she decompensated into shock. Broad-spectrum antibiotics were initiated when the patient went into shock but not at admission. It would have been prudent to start much earlier on the course, preferably upon initial evaluation. In cases like this, especially when an infectious etiology has not been fully excluded or there is clinical deterioration such as the development of CS, shock, or end-organ failure, initiation of broad-spectrum empiric antibiotics is highly recommended to curtail any possible infection and avert potential progression to septic shock.

Our patient was diagnosed with PCD based on the characteristic presentation of acute onset severe pain, edema, and cyanosis of the affected limb, coupled with imaging evidence of extensive DVT. Doppler ultrasound is a vital diagnostic and often sufficient modality for identifying and determining the extent of thrombosis in the deep and superficial veins. Further imaging, including venography and/or arteriography, may be necessary to elucidate the possible etiology or exclude any lesions that might predispose to DVT, as well as exclude possible underlying arterial thrombosis [1]. In our patient, Doppler ultrasonography demonstrated the extensive iliotibial DVT, and the abdomino-pelvic CTA revealed an iliac chain adenopathy along the right pelvic sidewall abutting the common iliac artery bifurcation, compressing and effacing the right external iliac vein likely precipitating the right lower extremity DVT.

Risk factors for PCD include malignancy, occluded inferior vena cava filters, and other prothrombotic states such as hormone replacement or contraceptive use, pregnancy, trauma or long-distance travel [1,9]. The left lower extremity is four times more likely affected than the right since the left common iliac vein is more commonly compressed by the overlying left common iliac artery, predisposing to thrombosis. In contrast, this is uncommon on the right as the right common iliac vein ascends vertically into the inferior vena cava [10]. Our patient’s history of breast cancer and the use of oral contraceptives, coupled with the compression of the right iliac vein by the iliac chain adenopathy - possibly a sequela of her uterine fibroids, likely predisposed her to developing extensive right lower extremity DVT and PCD.

Given its rarity, the therapeutic approach to the management of PCD is not well established. The mainstay of therapy, however, includes anticoagulation, fluid resuscitation, and systemic or catheter-directed thrombolysis (CDT) or surgical thrombectomy [1,11-12]. Ultimately, the goal is prompt clot removal to restore venous patency, limb salvage, and prevention of thrombose recurrence. Acute anticoagulation with UFH or low molecular weight heparin (LMWH) should be started immediately, whether or not thrombolysis is planned [13]. Our patient was promptly started on UFH since she was also anticipated to be taken to surgery and possible thrombolysis/thrombectomy. Although not widely studied, the novel oral anticoagulants (NOACs) including apixaban, have been successfully used to treat cancer-related PCD patients with no recurrence of thrombosis at six months [14,15]. While management with NOACs is studied for cancer-related VTE, it might not be appropriate for PCD management acutely, especially post-thrombolysis/thrombectomy given reversibility-related issues with NOACs. It is preferable to use a short-acting and readily reversible parenteral agent (UFH or LMWH) before and after lysis, after which the patient can be transitioned to a NOAC or warfarin later in the hospital course and for outpatient management. 

Thrombolysis or thrombectomy usually accompanies anticoagulation, as studies have shown that thrombolysis results in more rapid and complete lysis of clots compared to anticoagulation alone [16]. The decision to use thrombolysis (systemic versus catheter-directed measures) or surgical thrombectomy is largely dependent on the available resources, clinician expertise, and patient-specific factors [13]. CDT has higher rates of clot removal compared to thrombectomy alone [17]. Similarly, CDT is associated with a lower likelihood of bleeding than systemic thrombolysis, which is rarely used nowadays [18]. Pharmacomechanical thrombolysis (PMT) is a preferred treatment modality that combines mechanical thrombolysis modalities such as angioplasty or clot aspiration with the use of a pharmaceutical agent for local thrombolysis. Lin et al. [19] were able to show that PMT was better than CDT alone and led to an overall reduction in the length of ICU stay. Although thrombolysis/thrombectomy was planned for the patient after the fasciotomy, she unfortunately deteriorated and went into shock with multiorgan failure, further precluding additional interventions. We believe a faster approach to thrombolysis/thrombectomy, possibly done concurrently with the fasciotomies if feasible, may have improved the overall outcome of the patient.

Extensive lower extremity DVT resulting in PCD can be complicated by acute compartment syndrome, necessitating an emergent fasciotomy to prevent irreversible muscle necrosis and limb loss. Time is exigent in these situations as the outcomes can be very poor. Some studies have reported the need for subsequent limb amputations despite timely emergency fasciotomies. Although rare, the development of multiorgan failure resulting in death in PCD patients has been reported [1,4]. Our patient falls in the latter category. Even though she underwent an emergency fasciotomy with no evidence of muscle necrosis, her condition was compounded by the development of circulatory shock with multiorgan failure that culminated in her eventual death. The pathophysiology of the patient’s shock was likely multifactorial: hypovolemia due to blood loss during the fasciotomies (there was a reported EBL of 350 ml, but it is possible she bled more after the surgery) and distributive effects from possible underlying sepsis as well as from the local and systemic pro-inflammatory consequences of reperfusion and rhabdomyolysis, all resulting in tissue hypoperfusion, metabolic acidosis, and hyperkalemia. Nevertheless, a cardiogenic or obstructive component to the shock cannot be excluded especially as the patient never had an echocardiography or pulmonary imaging to exclude a pulmonary embolism. The ventricular fibrillation and cardiac arrest were likely precipitated by acidosis and hyperkalemia.

## Conclusions

PCD is a rare condition with high morbidity and mortality, often presenting as a triad of severe pain, swelling, and cyanosis of the affected limb with extensive DVT. Its recognition warrants timely and aggressive intervention with anticoagulation, thrombolysis, or thrombectomy to prevent progression to venous gangrene with possible limb loss or death. Heparin remains the anticoagulant of choice for acute treatment. Early recognition and treatment of PCD can prevent the development of CS by mitigating the early propagation of thrombosis. Right lower extremity involvement due to iliac vein compression is much less common than disease on the left but can occur. Similarly, the development of CS with eventual limb amputation and death can occur even with emergent fasciotomy. While our patient had a negative outcome, our case adds to the current literature and highlights the importance of identifying and treating PCD, as it is reversible if the patient undergoes appropriate therapy early. This case also highlights the role of antibiotics especially in the initial stages of management when an infectious etiology cannot be excluded.
